# Effects of solvent additive on “s-shaped” curves in solution-processed small molecule solar cells

**DOI:** 10.3762/bjoc.12.249

**Published:** 2016-11-28

**Authors:** John A Love, Shu-Hua Chou, Ye Huang, Guilllermo C Bazan, Thuc-Quyen Nguyen

**Affiliations:** 1 Center for Polymers and Organic Solids, University of California, Santa Barbara, California 93106, United States; 2 Institute of Physics and Astronomy, University of Potsdam, Potsdam-Golm 14476, Germany; 3 Department of Chemistry, National Taiwan University, Taipei, 10617, Taiwan

**Keywords:** current voltage analysis, morphology, organic solar cells

## Abstract

A novel molecular chromophore, p-SIDT(FBTThCA8)_2_, is introduced as an electron-donor material for bulk heterojunction (BHJ) solar cells with broad absorption and near ideal energy levels for the use in combination with common acceptor materials. It is found that films cast from chlorobenzene yield devices with strongly s-shaped current–voltage curves, drastically limiting performance. We find that addition of the common solvent additive diiodooctane, in addition to facilitating crystallization, leads to improved vertical phase separation. This yields much better performing devices, with improved curve shape, demonstrating the importance of morphology control in BHJ devices and improving the understanding of the role of solvent additives.

## Introduction

Tremendous multidisciplinary research efforts have led to consistent increases in the efficiency of organic solar cells, making the technology a bright prospect in the quest for alternative energy [1–4]. In particular the rapid development of solution-processed small molecule materials over the last several years has led to leaps in state-of-the-art efficiencies and improved understanding of structure–property relationships. The well-defined molecular structures have inherent amenability to purification, batch-to-batch reproducibility and monodispersity, which make them an attractive alternative to their polymeric counterparts [5–6]. Also of import stands the fact that easily modified, modular structures lead to finely-tunable energy levels and optical properties through molecular design [7–8]. Most high-performing small molecule electron-donor materials are configured such that the conjugated backbone consists of alternating electron-rich donor (D) and the electron-deficient acceptor (A) moieties so as to facilitate efficient photo-induced charge transfer and harvest a broad spectral response [9–11]. One such molecular architecture introduced by Bazan and co-workers can be described as a D_1_–A–D_2_–A–D_1_ system [12], where D_1_ is an electron-rich unit such as bithiophene, A is a benzothiadiazole derivative and D_2_ can be different electron-rich planar cores such as dithienosilol or silanindacenodithiophene. Utilizing this push–pull molecular approach, efficiencies up to 9.0% have been achieved [13] due to deep highest occupied molecular orbitals (HOMO) and the corresponding large open circuit voltages (*V*_OC_). There remains, however, room for improvement by further tuning the energetics of these materials so as to harvest photons from the widest possible spectral range while still maintaining high *V*_OC_. Expressly, the optical bandgap must be further reduced by minimizing the energetic gap in the lowest occupied molecular orbitals (LUMO) between donor and acceptor [14].

**Figure 1:**
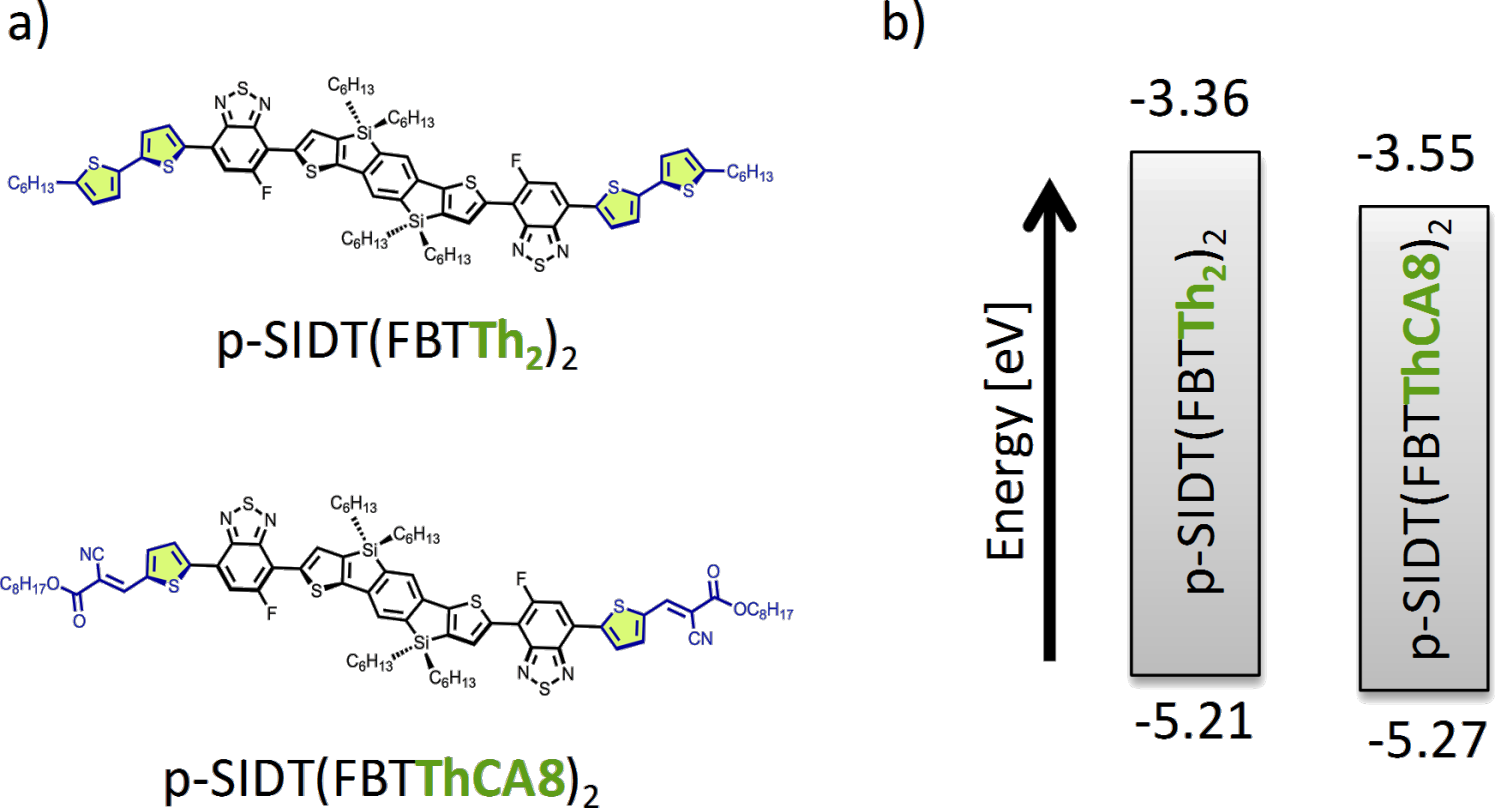
a) Molecular structures and b) energy levels of p-SIDT(FBTTh_2_)_2_ and p-SIDT(FBTThCA8)_2_ highlighting the modification of the end groups.

One design approach towards this end involves adding electron-withdrawing end groups to existing central core chromophores. Chen and co-workers have successfully used cyanoacetate [15] and other electron-withdrawing end groups to create A–D–A type oligothiophene derivatives with tunable electronics, which are among the highest performing materials to date [16–19]. Starting with the previously reported molecule benzo[1,2-*b*:4,5-*b*]bis(4,4′-dihexyl-4*H*-silolo[3,2-*b*]thiophene-2,2′-diyl)bis(6-fluoro-4-(5′-hexyl-[2,2′-bithiophene]-5-yl)benzo[*c*][1,2,5]thiadiazole, p-SIDT(FBTTh_2_)_2,_ (Figure 1), we have modified the conjugated backbone to include electron-withdrawing octyl cyanoacetate (CA8) end groups, essentially forming an “A_1_–D_1_–A_2_–D_2_–A_2_–D_1_–A_1_” molecular skeleton, benzo[1,2-*b*:4,5-*b*]bis(4,4′-dihexyl-4*H*-silolo[3,2-*b*]thiophene-2,2′-diyl)bis(6-fluoro-4-((*E*)-octyl-3-(5-thiophen-2-yl)-2-cyanoacrylate]-5-yl)benzo[*c*][1,2,5]thiadiazole, p-SIDT(FBTThCA8)_2_. We will show that this molecular substitution did indeed significantly reduce the bandgap while maintaining deep energy levels, as well as some of the other desirable properties of the parent material [20–22].

However, molecular design remains only the first step towards the development of high performance devices. Mismanagement of the organization and phase-separation processes or inappropriate device architecture choices can lead to non-ideal electronics at electrode interfaces and severely limit the performance of a materials system. In particular, the fill factor (*FF)*, which is simply a metric describing the field dependence of current, can be particularly sensitive to changes in morphology or interfacial effects. At the extreme, a strong field dependence near open circuit conditions can result in current vs voltage (*J–V*) curves adopting an “s-shape.” Such curves have been observed experimentally in a number of diverse OPV systems, and have been ascribed to a host of morphological or interfacial issues. The s-shape arises from inefficient charge extraction of one charge carrier type at small electric fields. Mechanistically, a variety of underlying causes have been proposed, including large imbalances in charge carrier mobilities, energetic barriers to charge extraction at electrode interfaces, reduced surface recombination, and interfacial defects leading to traps; device simulations have shown that all of these could indeed result in the s-shape behavior [23–30].

Herein we describe the development of a novel small molecule system with nearly ideal optoelectronic properties, which unfortunately results in s-shaped *J–V* curves and poor performance. We show that this is due to non-ideal phase separation, specifically a preferential migration of the electron acceptor to the bottom anode interface. This can, however, be mitigated through appropriate processing, using a small amount of the solvent additive 1,8-diiodooctane (DIO).

## Results and Discussion

### Synthesis and characterization

**Scheme 1:**
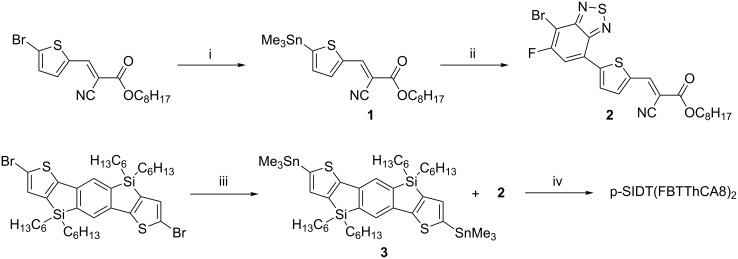
Synthetic route towards p-SIDT(FBTThCA8)_2_. (i) Sn_2_Me_6_, Pd(PPh_3_)_4_, toluene, 85 °C; (ii) 4,7-dibromo-5-fluorobenzo[*c*][1,2,5]thiadiazole, Pd(PPh_3_)_4_, toluene, 90 °C, 42.8%; (iii) *n*-BuLi, THF, −78 °C, 10 min; then Me_3_SnCl, 98.9%; (iv) Pd(PPh_3_)_4_, toluene, MW, 80.6%.

Scheme 1 depicts the synthesis toward p-SIDT(FBTThCA8)_2_. As opposed to using bottom-up synthetic procedures as reported in the literature in which ketone derivatives are converted to octyl cyanoactates in the final synthetic step via Knoevenagel condensation [15,31–35], we chose to begin with (*E*)-octyl 3-(5-bromothiophen-2-yl)-2-cyanoacrylate as the starting material to ensure good solubility. Intermediate **1** was prepared by palladium-mediated stannylation [36] and then subjected to regioselective Stille conditions [37] in an oil bath (90 °C) to generate **2**. (Complete synthetic details and characterization of all compounds are provided in Supporting Information File 1). Its fluorine regiochemistry was confirmed by ^1^H-^1^H NOESY spectroscopy (Supporting Information File 1, Figure S1). Compound **3** was obtained through lithium–halogen exchange with *n*-BuLi followed by addition of trimethyltin chloride. Segments **2** and **3** were cross-coupled using a microwave-assisted Stille reaction to afford the target p-SIDT(FBTThCA8)_2_.

The thermal transitions of p-SIDT(FBTThCA8)_2_ were evaluated by differential scanning calorimetry (DSC) and compared to its predecessor, p-SIDT(FBTTh_2_)_2_. A significant impact on thermal behaviors was observed upon substituting 2-hexylthiophene with octyl cyanoacetate (Supporting Information File 1, Figure S4 and Table S1). As compared to p-SIDT(FBTTh_2_)_2_, the melting (*T*_m_) and crystallization (*T*_c_) temperatures of p-SIDT(FBTThCA8)_2_ are increased by 76.9 and 117.1 °C, respectively, which implies an enhancement of the intermolecular interaction in the solid state. This improved rigidity of p-SIDT(FBTThCA8)_2_ is correlated to a noticeable decrease in solubility, which was measured to be 32 mg/mL for p-SIDT(FBTThCA8)_2_ compared with over 50 mg/mL for p-SIDT(FBTTh_2_)_2_ in chloroform at room temperature.

Frontier molecular energy levels were estimated by cyclic voltammograms (CV) in dichloromethane and calculated theoretically by density functional theory (DFT) (Supporting Information File 1, Figure S5 and Table S2). In the CV measurement, energy levels of HOMO and LUMO were calculated from the onsets of oxidation and reduction potentials. The HOMO level (*E*_HOMO, CV_: −5.27 eV, *E*_HOMO, DFT_: −5.43 eV) is quite deep, even compared to that of p-SIDT(FBTTh_2_)_2_ (*E*_HOMO, CV_: −5.21 eV, *E*_HOMO, DFT_: −4.97 eV). We anticipate this should provide a high *V*_OC_ when blended with PCBM. The band gap of p-SIDT(FBTThCA8)_2_ is also reduced with respect to p-SIDT(FBTTh_2_)_2_ as determined by CV (1.72 eV and 1.85 eV, respectively) and by DFT (1.90 eV and 2.01 eV, respectively) suggesting that substituting 2-hexylthiophene with octyl cyanoacetate on both wing-ends does noticeably reduce the bandgap while maintaining a deep HOMO level.

**Figure 2:**
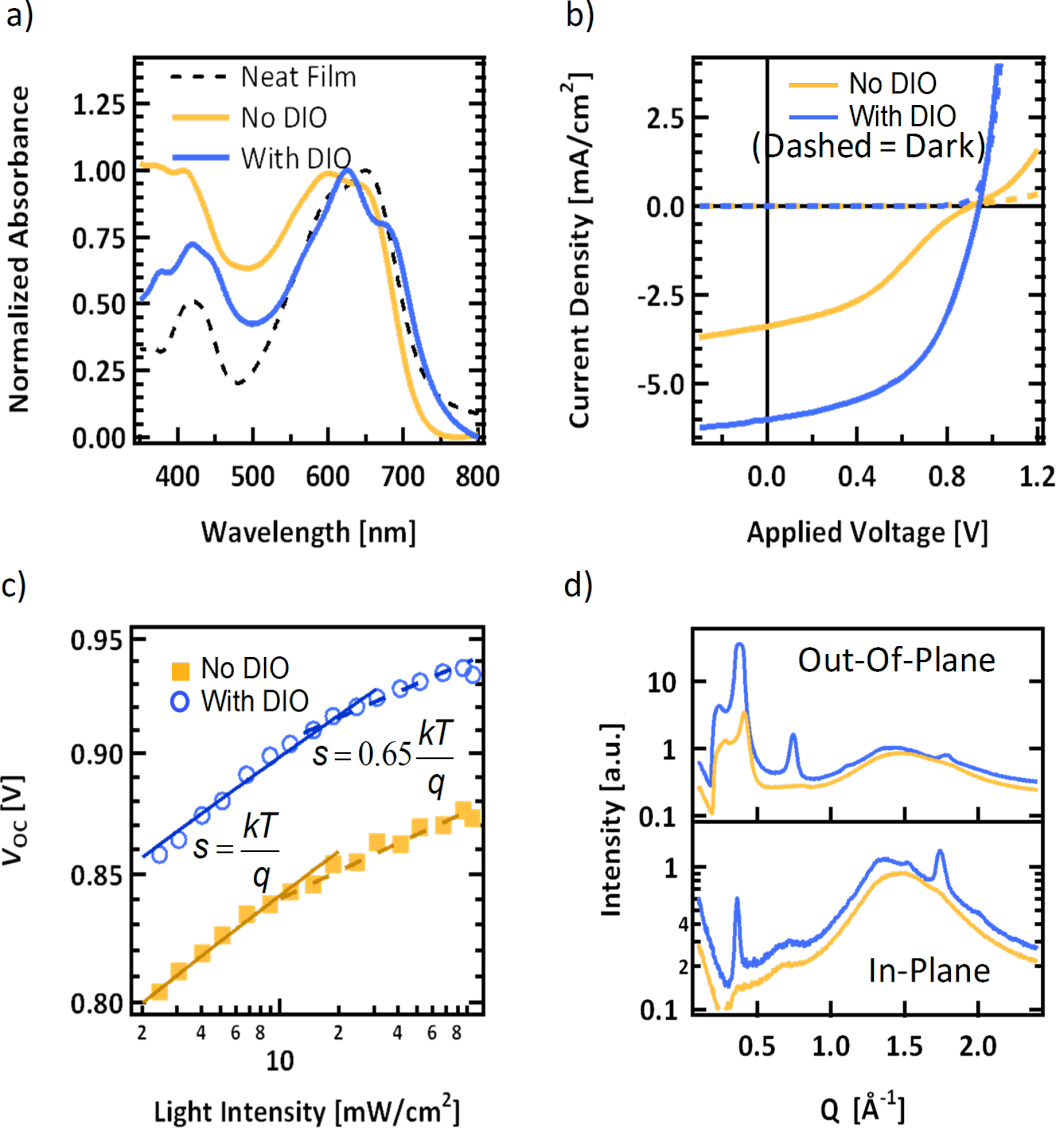
a) Solid-state absorption profiles of neat p-SIDT(FBTThCA8)_2_ (dashed line) and p-SIDT(FBTThCA8)_2_:PC_71_BM blends cast from pure chlorobenzene (yellow) and with 1.5% DIO (blue). b) Photovoltaic performance of equivalent blend solar cells with c) corresponding light intensity open circuit voltage measurements where the empirically fit solid lines have a slope of *kT*/*q* and dashed lines indicate a slope of 0.65 *kT*/*q*, d) blend film X-ray diffraction line cuts from crystallites oriented out-of-plane (top) and in-plane (bottom).

The normalized solid-state absorption profile of p-SIDT(FBTThCA8)_2_ is shown as the dotted line in Figure 2a and the data are also summarized in Table S1 (Supporting Information File 1). The film has strong absorption in the visible range, with an onset at 750 nm corresponding to an optical bandgap of 1.65 eV. This is consistent with the electrochemically determined bandgap. The primary absorption band shows vibronic progression, suggesting ordering in the solid state, with peak absorption at 650 nm. The red-shifted absorption of p-SIDT(FBTThCA8)_2_ with respect to p-SIDT(FBTTh_2_)_2_, whose absorption onset in the solid state occurs at 670 nm, is further confirmation that the addition of electron-withdrawing endgroups reduces the bandgap of the chromophore. Importantly, the shift in absorption onset represents about a 25% increase in the number of photons in the AM 1.5 solar spectrum available for absorption. If p-SIDT(FBTThCA8)_2_ maintains high internal quantum efficiencies and *FF* like its predecessor, and also achieves a high *V*_OC_ as expected based on energy levels, the improved absorption imparts p-SIDT(FBTThCA8)_2_ with great potential.

### Solar cell performance

For initial photovoltaic device fabrication, conditions were chosen according to previously reported protocols of structurally similar small molecule systems [38–40]. Specifically, p-SIDT(FBTThCA8)_2_ was mixed with PC_71_BM and cast to form a bulk heterojunction (BHJ) atop poly(3,4-ethylenedioxythiophene) polystyrene sulfate (PEDOT) giving an architecture of ITO/PEDOT/p-SIDT(FBTThCA8)_2_:PC_71_BM/Ca/Al. The mass ratio of p-SIDT(FBTThCA8)_2_:PC_71_BM was held at 1:1 and cast from a chlorobenzene solution containing 40 mg/mL total solids, giving 120 nm thick active layers. Such devices show modest performance (*J*_SC_ = 3.4 mA/cm^2^, *V*_OC _*=* 0.91 V, *FF* = 0.37, *PCE* = 1.1%). Though the performance is low, the efficiency is similar compared to other systems cast from pure chlorobenzene. Furthermore, the high *V*_OC_ of 910 mV is encouraging, as it further confirms the advantage of the deep lying HOMO level of p-SIDT(FBTThCA8)_2_. However, an inflection point near *V*_OC_, a clear kink in the *J–V* curve gives the curve a dramatic “s-shape” (Figure 2b) limiting *FF* and *PCE_._*

In the literature, it has been shown that incorporation of small amounts of the solvent additive DIO into the casting solvent can vastly improve small molecule device performance [38–43]. Accordingly, initial optimization required adjusting the concentration of DIO. It was found that at a concentration of 1.5% DIO (by volume) in chlorobenzene, the *PCE* was increased to 2.9% (*J*_SC_ = 6.0 mA/cm^2^, *V*_OC_ = 0.94 V, *FF* = 0.52); device characteristics are shown in Table 1. Though, the improvements in device performance are relatively modest compared to what has been observed in other systems, incorporation of the DIO into the solution noticeably reduces the s-shape of the curve leading to a greatly enhanced *FF*. While the use of additives has been shown to have a number of consequences on film formation and device operation [38–44], to the best of our knowledge, such a dramatic change in curve shape has not been demonstrated previously using solvent additives. And while these additive-processed devices still have not nearly reached the full potential of this materials system, and other possible processing changes may also affect the nature of the *J–V* curve, we have focused herein on understanding the mechanism leading to the change in curve shape to gain a better, fundamental understanding of the nature and operation of small-molecule solar cell devices and the role of solvent additives in film formation.

**Table 1:** Device characteristics when cast with and without DIO, before and after treatment with MeOH in a standard architecture as well as in an inverted cell.

		Solar cell characteristics			

Conditions		*J*_SC_ (mA/cm^2^)	*V*_OC_ (V)	*FF*	*PCE* (%)

No DIO	standard	3.4	0.91	0.37	1.1
	w/MeOH	3.4	0.95	0.37	1.2
	inverted	4.5	1.09	0.51	2.5
With DIO	standard	6.0	0.94	0.52	2.9
	w/MeOH	6.1	1.02	0.52	3.2
	inverted	7.0	0.73	0.47	2.4

As a first insight into the difference in *J–V* behavior with and without DIO we examined the light intensity dependence of the two devices. Varying the intensity of the incident light serves to proportionally change the number of absorbed photons and thus generation of free charges. Of particular interest is the effect of light intensity on *V*_OC_, since at the open circuit voltage carriers are created, but nearly none of the charges are extracted, *J =* 0; all charges must therefore recombine [45]. Thus, the relation of *V*_OC_ with the incident light intensity for bimolecular (free charge) recombination has been shown to depend only on temperature and light intensity, given by

[1]
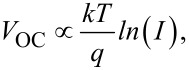


where *I* is light intensity, *k* is the Boltzman constant, *T* is temperature and *q* is the elementary charge. Thus, in a system dominated by bimolecular recombination, on a semi-log plot of *V*_OC_ vs *I* we expect a linear relationship with a slope of *kT/q* [45]*.* It is worth noting that proper analysis of low light intensity data requires sufficiently low dark current, such that it does not constitute a significant fraction of the device current in the voltage regime close to *V*_OC_. In both the devices cast with and without additive, even at only 0.02 suns, the dark current remains at least two orders of magnitude lower than the device current (see Supporting Information File 1, Figure S6). The *V*_OC_ as a function of light intensity are shown in Figure 2c for devices without and with DIO.

It is immediately clear that the *V*_OC_ in devices without additive do not follow a single linear relationship across all light intensities. Instead it seems to follow a slope of *kT/q* closely at light intensities lower than 10 mW/cm^2^, but then has a shallower, seemingly linear dependence with a slope of ≈0.65 *kT/q* at higher intensities. The slope of 0.65 *kT/q* was fit empirically and does not fit the data unequivocally, but is displayed to show at the very least, that at higher light intensities the *V*_OC_ has a dependency that is less than the expected *kT/q.* The suggestion is that at high charge densities, the dominant recombination mechanism may change. The device cast with DIO shows similar behavior but to a much lesser extent. The *V*_OC_ only deviates from *s = kT/q* significantly at intensities close to 100 mW/cm^2^. Thus, even devices processed with DIO may, to some extent, suffer from the same problems as those cast from pure chlorobenzene albeit to a much lesser extent. Light intensity studies are thus a powerful tool to look at more nuanced details of current voltage characteristics.

**Figure 3:**
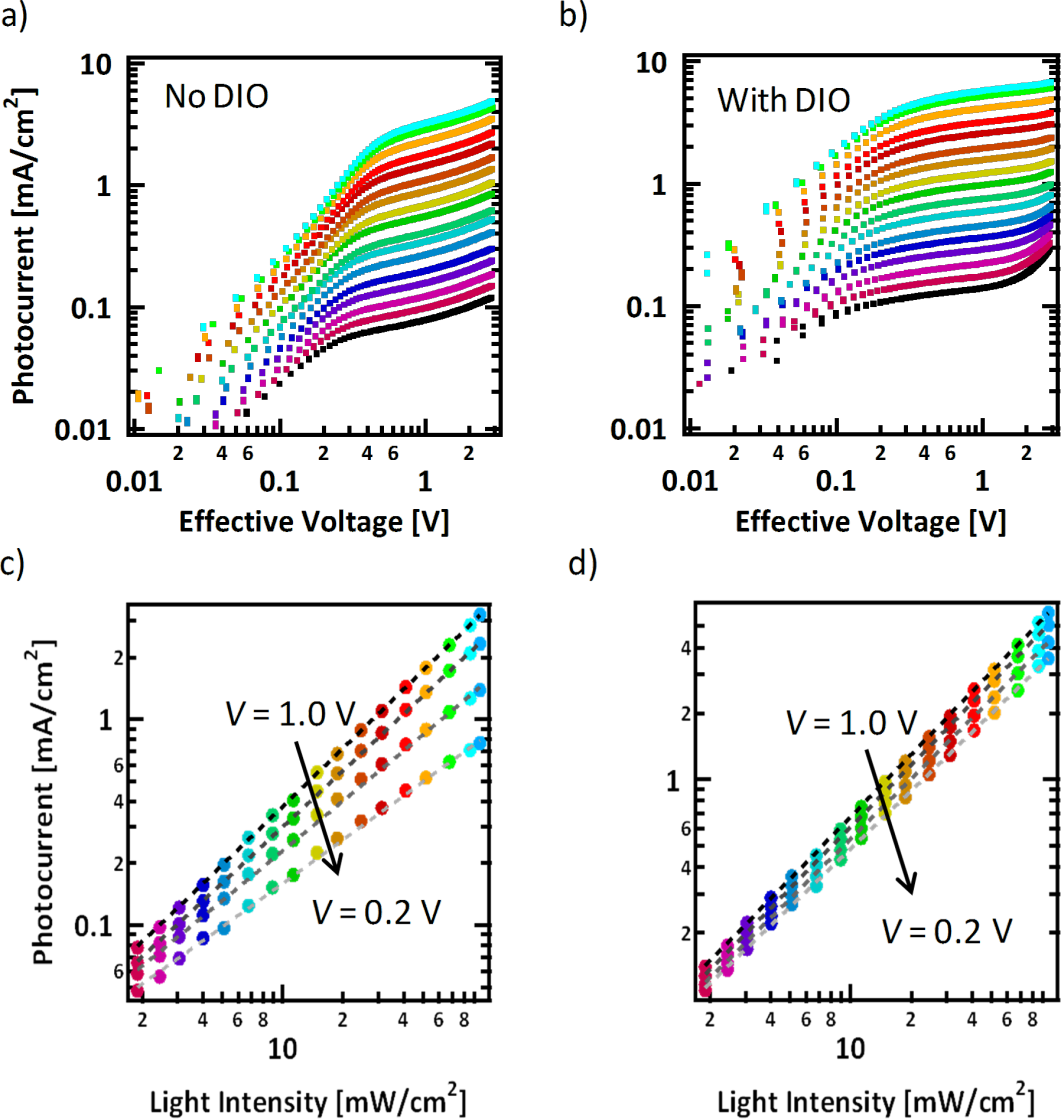
Light intensity dependence of photocurrent as a function of the effective voltage, *V*_0_* − V,* for devices cast a) without DIO and b) with DIO and the extracted photocurrent at effective voltages of 1.0, 0.5, 0.3, and 0.2 V (from black to grey, respectively) as detailed in Table 2 for devices cast c) without DIO and d) with DIO.

To further inspect the effects of light intensity on device operation, the photocurrent, *J*_Ph_*,* defined as the current upon illumination with the dark current subtracted, was examined as a function effective voltage [46–49]. The effective voltage is the voltage difference between the applied voltage and the voltage at which no photocurrent is generated, *V*_0_* − V*, and determines the strength of the electric field within the device, the driving force for charge extraction. *J*_Ph_ is shown as a function of light intensity for devices cast without and with DIO in Figure 3a and 3b, respectively.

At low effective voltages, (*V*_0_* − V* < ≈0.1 V) implying a small electric field, the photocurrent of both devices linearly increases with voltage. This is due to the competition between drift and diffusion of photogenerated charges to the contacts [49]. In the device processed with DIO, beyond *V*_0_* − V* = 0.2 V the *J*_Ph_ reaches a saturation regime, where it increases much less significantly with voltage. In this saturation regime, the larger electric field can effectively sweep out charges and bimolecular recombination does not play as significant a role. The voltage at which this rollover point occurs is independent of intensity. In these devices, there is not a true “saturation” as the photocurrent is always increasing, however, there is still a clear rollover point between two regimes. This increasing photocurrent could be due to field dependent charge generation [50–53].

As seen in Figure 3a, *J*_Ph_ has a much stronger dependence on voltage in devices processed without DIO. Even at high effective voltages, there remains a strong voltage effect and *J*_Ph_ continues to increase without saturating. There are two clear regimes with two different voltage dependencies, but in contrast to devices processed with DIO, in this case the rollover voltage at which *J*_Ph_ switches from one regime to the other does indeed depend on light intensity. At higher intensities, a higher voltage is required to reach the “saturation” regime. This has previously been associated with a build-up of space charge in the film [47].

It is expected that for devices not limited by charge extraction, *J*_Ph_ at each and every effective voltage, should scale linearly with intensity, *J*_Ph_ 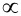 *I,* while devices limited by space charge build-up have been shown to characteristically have a sub-linear dependence, where *J*_Ph_ 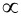 *I *^0.75^ [47]. At *V*_0_* − V* = 1.0 V, close to short-circuit conditions, in devices processed with and without additive, *J*_Ph_ scales nearly linearly, following a power law where s = 0.95. This relation deviates from linearity when moving to lower fields particularly in the devices cast without DIO. As seen in Table 2, at an effective voltage of 0.3 V, s = 0.81 and at 0.2 V, s = 0.71. This is quite close to 0.75, the value one would expect for a device limited by space charge.

**Table 2:** Power law dependences of photocurrent on light intensity at specific effective voltages for BHJ devices from Figure 3.

Conditions	Power law dependence			
	0.2 V	0.3 V	0.5 V	1.0 V

no DIO	0.71	0.81	0.91	0.95
with DIO	0.88	0.91	0.94	0.95

In Figure 3b, a pronounced uptick in photocurrent is seen at high reverse biases (>1.5 V). This, however, is likely an artifact, as the “photocurrent” seems to follow the dark current which is not as low in the additive processed film as in the film without DIO. The dark current is plotted with the light intensity studies in Figure S6 (Supporting Information File 1). While in the photocurrent the dark current is subtracted from, the illuminated it is likely that the linear leakage current may also change with light. This highlights the need for low levels of leakage current for reliable measurements at higher voltages.

At low fields, the device processed without DIO suffers from space charge build-up, while at higher fields, there is sufficient driving force to overcome these effects and extract the charges. A similar effect can be seen in the device processed with DIO, albeit to a lesser extent. At *V*_0_* − V* = 0.2 V in the optimized device, s = 0.88. This suggests again that while the DIO does not completely remove problems associated with charge extraction, it significantly reduces the magnitude of the effects, removing the dramatic s-shape of the curve.

### Thin film X-ray diffraction

Changes in device performance upon addition of solvent additives are typically ascribed to improvements in the BHJ nanostructure by affecting the thermodynamics and kinetics of phase separation. In this class of small molecule systems, this is often attributed to asserting control over the crystallization and phase-separation processes within the blend; DIO helps induce crystallinity of the donor material [40–42,54–56]. Grazing incidence wide-angle X-ray scattering (GIWAXS) was used to probe the crystallization behavior of the blend system with and without additive. The full 2-dimensional GIWAXS spectra from a film of the neat p-SIDT(FBTThCA8)_2_ and the two blends are shown in Figure S7 (Supporting Information File 1) while line cuts showing *Q*_z_ (“out-of-plane”) and *Q*_x-y_ (“in-plane”) of the two blends are shown in the top and bottom plots respectively of Figure 2d.

Looking first at the out-of-plane diffraction in the top panel of Figure 2d, the BHJ film cast with no DIO shows a prominent peak at 0.37 Å^−1^. This corresponds to a real-space distance of 1.7 nm. While attempts to grow single crystals of this material have thus far been unsuccessful and thus the peaks cannot be indexed precisely, by convention we attribute this spacing to an “alkyl stacking peak”, that is a spacing arising from molecules separated by alkyl chains analogous to the lamellae stacking in P3HT (i.e., (100) planes). In the film cast with DIO, this peak is more prominent suggesting a greater degree of crystallinity. There is also a peak at 0.74 Å^−1^, which corresponds to the second order reflection. There is even a small peak at 1.11 Å^−1^, which likely corresponds to a third-order reflection, suggesting a quite well-ordered film. Additionally, there is a small peak at 1.79 Å^−1^, corresponding to a spacing of 3.5 Å, which we attribute to π–π stacking. There is a broad feature centered at *Q* = 1.5 Å^−1^ which is seen in both films and at all orientations, which is the convolution of two peaks. In the neat p-SIDT(FBTThCA8)_2_ there is a relatively weak, broad feature at 1.52 Å^−1^ which convolves with the isotropic scattering peak of PC_71_BM which is typically found at 1.3–1.4 Å^−1^. These two peaks are nearly resolvable in the in-plane scattering of the film cast with DIO but are completely overlapping in the blend without additive, leading to a very broad peak.

Looking next at the traces from the *Q*_x-y_ direction, that is, just from crystallites oriented in the plane of the substrate, there are no discernible features from p-SIDT(FBTThCA8)_2_ in the BHJ film cast without DIO. In the film processed with DIO, the alkyl stacking peak is again though is less prominent in-plane, while the π-stacking peak is more prominent. Assuming the alkyl and π-stacking directions are perpendicular, this suggests the material primarily adopts an edge-on orientation. This is in contrast with the preferential “face-on” orientation adopted by p-SIDT(FBTTh_2_)_2_ [40], demonstrating how sensitive molecular self-assembly can be to relatively small molecular design choices. However, consistent with previous reports of related molecules, DIO does seem to improve crystallinity.

Atomic force microscopy (AFM) topography images are shown in Figure S8 (Supporting Information File 1). The film cast without DIO has a relatively smooth featureless surface while the film cast with DIO has a rougher surface with relatively large (>100 nm diameter) features. This is consistent with phase separation and the crystallinity seen by GIWAXS.

Despite the differences in crystallization, this does not give a clear indication as to the root cause of why devices processed without DIO show signs of space charge and an s-shaped *J–V* curve. One might expect that the increase in crystallinity has a profound effect on the hole mobility in the blends, and space charge may occur due to imbalanced carrier mobilities in the device processed without DIO. However, the hole mobilities for blends processed without DIO and with 1.5% DIO are 5 × 10^−5^ and 9 × 10^−5^ cm^2^/Vs, respectively, each slightly lower than the neat hole mobility of p-SIDT(FBTThCA8)_2_, which is found to be 2 × 10^−4^ cm^2^/Vs (Supporting Information File 1, Figure S8). Although the mobility indeed increases with DIO processing, a mobility increase by a factor of two is not particularly significant and should not lead to such drastic changes in curve shape [24,30,57]. These mobilities are, however, somewhat lower than in related high-performance systems, which may always limit the system to a relatively low *FF* [40,57–58]. Unfortunately attempts to measure electron mobilities in charge-selective diodes were unsuccessful due to poor film formation on aluminum bottom contacts.

### Origin of the s-shape in *J–V* curves

**Figure 4:**
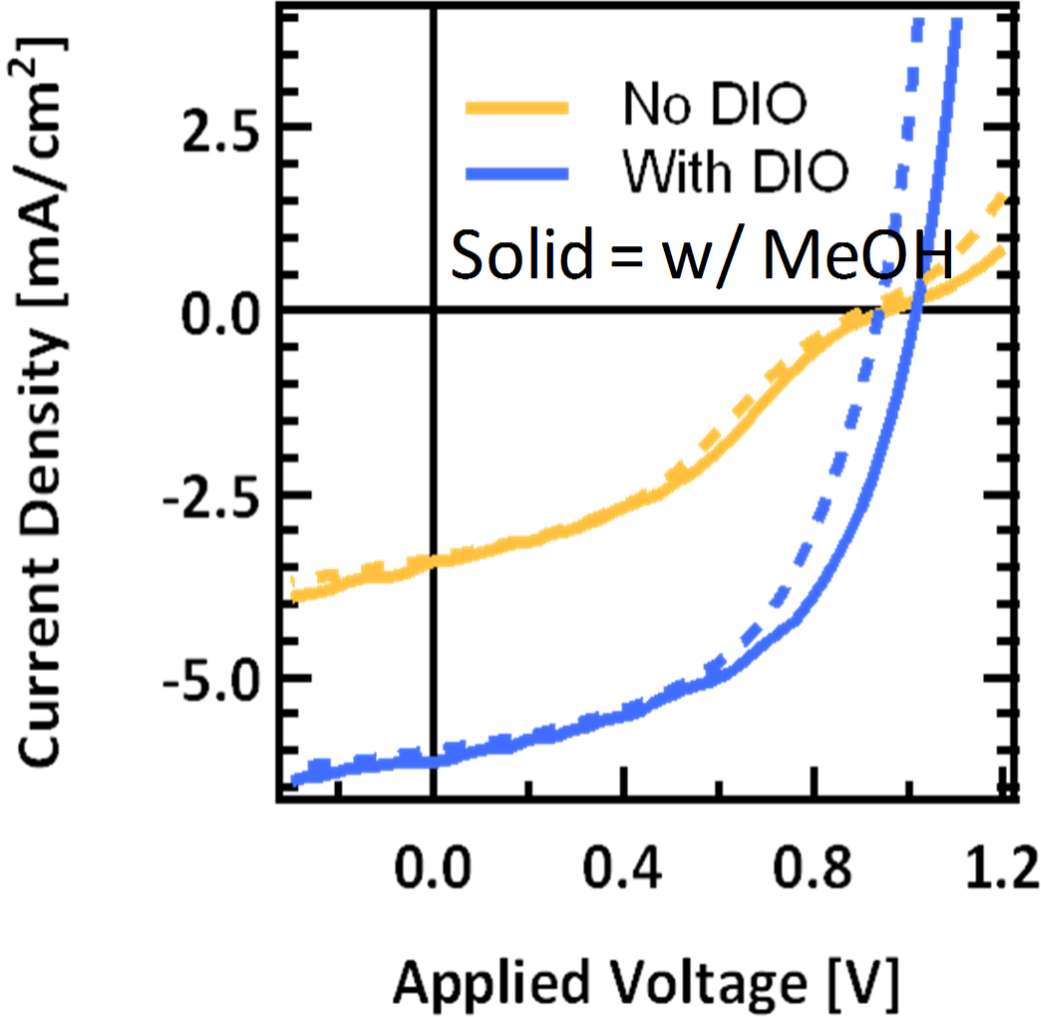
Current voltage curves for devices cast from pure chlorobenzene (yellow) and with 1.5% DIO (blue) with (solid) and without (dashed) methanol treatment.

Despite the relatively high *V*_OC_, based on the CV data, one might expect to achieve voltages that are even higher compared with p-SIDT(FBTTh_2_)_2_, as p- SIDT(FBTThCA8)_2_ seems to have an even deeper HOMO level. However, a HOMO of −5.27 eV is close to the work function of the PEDOT interfacial layer, and thus there may be non-ohmic contacts between the PEDOT and active layer, limiting the voltage [59]. Such an extraction barrier may also explain the build-up of space charge at one contact, and the s-shape to the *J–V* curve [25,30,60–62]. It has recently been shown by Tan and co-workers that in some cases, when PEDOT limits the voltage in solar cells, casting methanol on top of the layer will improve efficiency [63]. The methanol has been shown to effectively deepen the work function of the anode layer while not significantly disrupting the morphology. Specifically, this improves the extraction rate of holes at the anode interface. An enhanced hole extraction rate at the semiconductor/anode interface will reduce the accumulation of holes near the electrode, thereby preventing the screening of the internal field and suppressing recombination. The reduction of charge recombination and improved transport enables a higher photocurrent collection yield across the forward bias regime and improved *V*_OC_ [63]. We employed this processing method to improve the voltages in p-SIDT(FBTThCA8)_2_:PC_71_BM cells and look at the effects on curve shape (Figure 4).

After treatment with methanol, the *V*_OC_ of devices processed with DIO increases to 1.01 V. A similar improvement in *V*_OC_ is also seen for devices cast from chlorobenzene. The *J–V* characteristics are described in Table 1 and shown in Figure 4 along with the *J–V* curves replotted from Figure 2b for comparison. Treatment with methanol has a little effect on *J*_SC_ or *FF;* thus we suspect there is no significant change in morphology when methanol is cast. Rather, the treatments strictly improves electrical contact by deepening the work function as described previously [28,34,60–62]. Despite the improvement in *V*_OC_ in both devices, for devices processed without additive, the s-shaped kink in the *J–V* curve near open circuit remains. Thus, a contact problem at the anode is ruled out as the underlying cause of the atypical curve shape.

**Figure 5:**
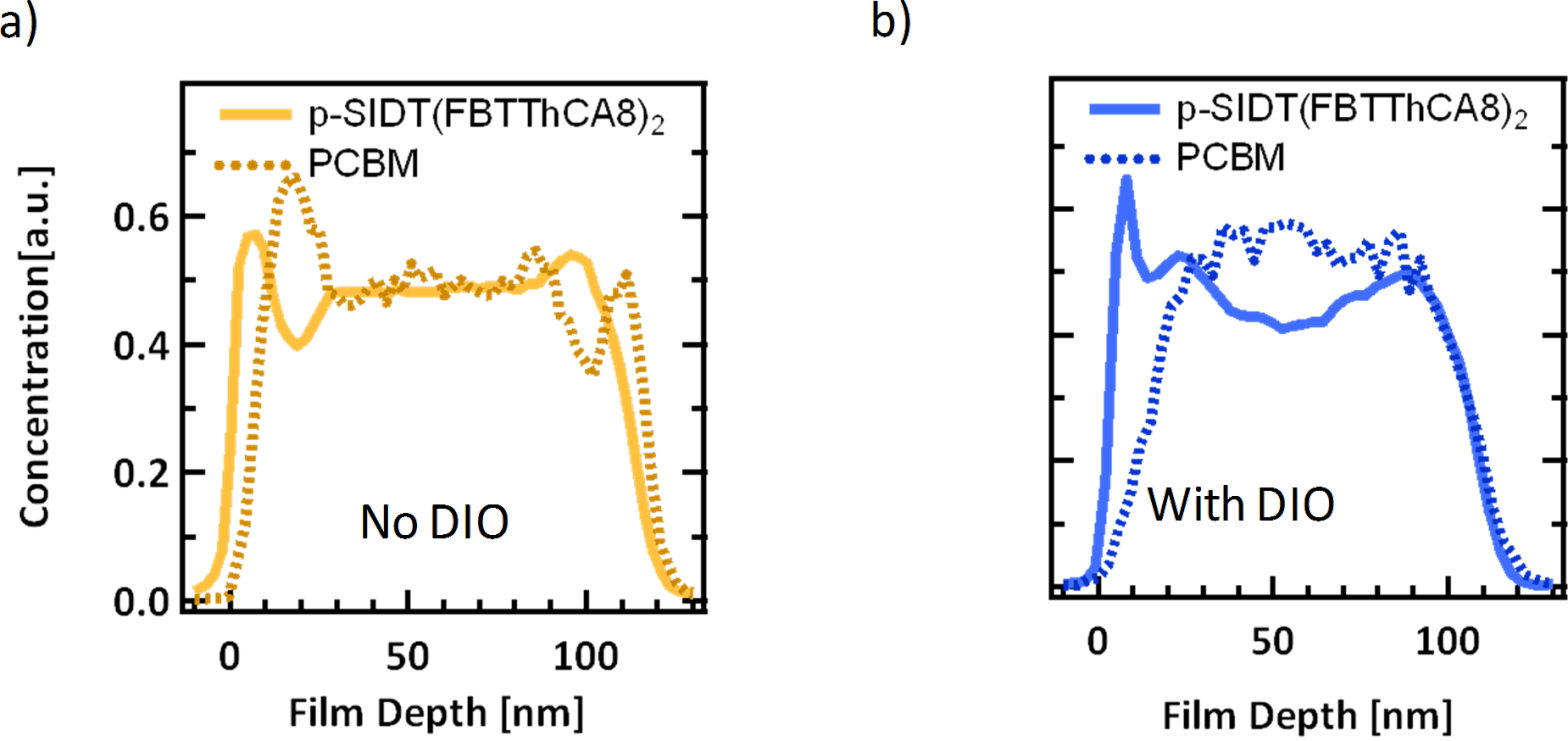
Dynamic secondary ion mass spectrometry (DSIMS) profile showing scaled nitrogen (solid) and deuterium (dashed) signals for films cast a) with no DIO and b) with 1.5% DIO.

Non-ideal vertical phase separation, that is to say, enrichment of donor material at the cathode or acceptor at the anode may also be a potential cause of s-kinks in *J–V* curves. The acceptor material at the PEDOT interface, for instance, can act as a barrier to hole extraction, leading to ineffective sweep out and a build-up of holes [23,26,64]. To examine the vertical separation behavior of the two blends dynamic secondary ion mass spectrometry (DSIMS) was employed. In DSIMS, a sample is bombarded with ions, ablating ionized material, which is analyzed using a mass spectrometer [65]. The composition of the ablated material is monitored as the beam mills through the thin film, resulting in a depth profile. To improve contrast between the two materials, deuterated fullerene PC_61_BM-*d*_5_ was used as a surrogate for PC_71_BM to establish a unique mass signal for the fullerene component [12,66–67]. Thus detection of deuterium in the mass spectrum implicitly signifies PC_61_BM-*d*_5_ in the film. The implicit assumption made here is that blends with the surrogate PC_61_BM-*d*_5_ behave phenomologically like those made with PC_71_BM, and thus the PC_61_BM-*d*_5_ signal will be applied to analyze the PC_71_BM-containing blend. The amount of p-SIDT(FBTThCA8)_2_ was monitored as the occurrence of nitrogen atoms in the mass spectrum. Unique signatures for each material help to make discerning relative concentrations simple and accurate. The DSIMS profiles of the two systems are shown in Figure 5.

As the DSIMS profile is collected, time corresponds to film depth, as the beam ablates material at a constant rate. Thus the x-axis has been scaled for film thickness, where the turn-on of the nitrogen and deuterium signals at *x* = 0 nm corresponds to the top surface of the films, what would be the cathode interface in a complete device architecture. The turn-off of the signals thus corresponds to the BHJ/PEDOT interface. The absolute intensity of the two signals given by the instrument cannot be compared directly due to different instrumental sensitivity, thus each signal is scaled to an average composition of 50% based on the weight ratio used in the blend solutions. It is fair to monitor how the signals evolve relative to each other as the beam penetrates into the film.

Looking first at the BHJ processed without additive, when the signals first turn on, there is initially an enrichment of p-SIDT(FBTThCA8)_2_ immediately followed by a depletion of donor and an enrichment of the PC_61_BM-d_6_ signal. This corresponds to donor material preferentially accumulated on the top surface. Throughout the bulk of the trace, the concentration of the two materials remains nearly constant. At the PEDOT interface, *x* = 115–120 nm, the PC_61_BM-*d*_6_ signal has a small peak while the p-SIDT(FBTThCA8)_2_ signal begins to drop off. This suggests that in the device there is an enrichment of PC_71_BM at the anode surface. Such an arrangement, with donor at the top surface and acceptor at the bottom, is non-ideal for the standard device architecture.

Processing with DIO has a significant effect on the vertical phase separation. At the top surface there is again an enrichment of the p-SIDT(FBTThCA8)_2_, evidenced by a faster turn on than the PC_61_BM-*d*_6_ signal. There is then a slight depletion of the p-SIDT(FBTThCA8)_2_ through the bulk of the device. At the bottom surface, however, unlike in the film cast without DIO, the two material signals overlap, suggesting an even distribution of p-SIDT(FBTThCA8)_2_ and PC_71_BM in the better performing devices. The vertical phase separation is still not ideal in this additive processed film, as there remains an enrichment of p-SIDT(FBTThCA8)_2_ at the cathode interface, however, DIO helps to overcome the problem of PC_71_BM concentrated at the anode interface.

A high concentration of PC_71_BM at the anode helps to explain the s-shape behavior of the *J–V* curve for the devices processed without additive. The low concentration of p-SIDT(FBTThCA8)_2_ near that interface reduces the surface recombination velocity of holes within the device; reduced surface recombination results in a piling up of charges near the anode which creates a space charge effect in the device [64]. This helps to explain the anomalous *V*_OC_ and *J*_Ph_ light intensity data. The effect is most apparent at low fields and high carrier concentrations, i.e., near open circuit conditions and at high light intensities.

If the s-shape seen in devices cast from chlorobenzene is in fact due to an enrichment of PC_71_BM at the bottom interface, the use of an inverted device architecture should result in an improvement in curve shape. The inverted architecture has the cathode as the bottom contact and the anode on top; thus if the vertical separation in the BHJ remains, the PC_71_BM-rich phase will be at the cathode interface and p-SIDT(FBTThCA8)-rich phase at the anode interface [68]. However, it is not necessarily true that the phase separation observed in one architecture will occur in inverted devices, as fabrication requires casting atop different substrates with different surface energetics, which may play a role in determining film formation.

**Figure 6:**
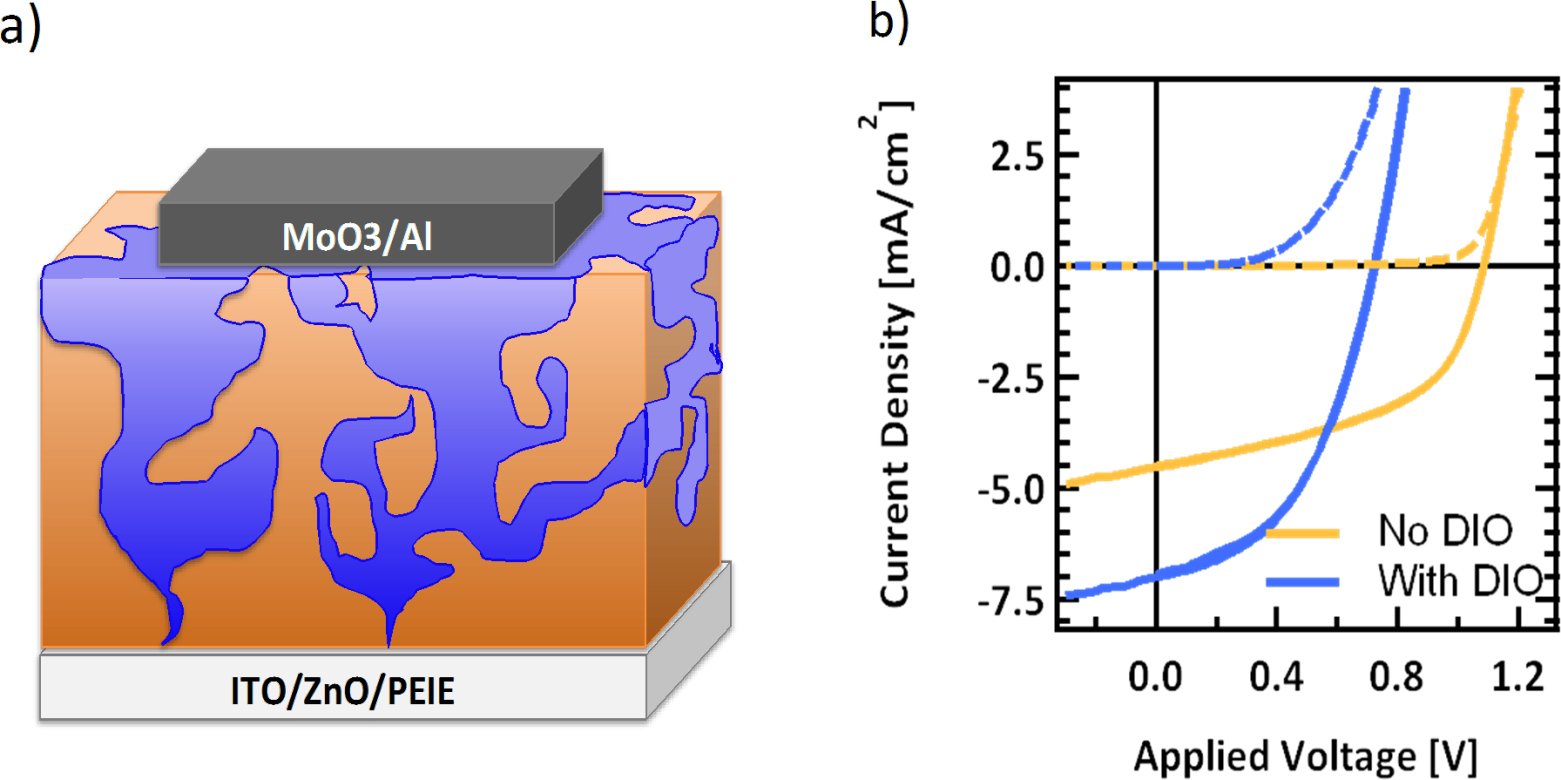
a) A schematic diagram of inverted architecture and b) *J–V* curves of device cast with no DIO in the standard (dashed) and inverted (solid) architecture.

While the active layers were cast in the same way, for inverted devices we employed the architecture ITO/ZnO/PEIE/p-SIDT(FBTThCA8)_2_:PC_71_BM/MoO_3_/Al where PEIE refers to ethoxylated polyethylenimine. The cathode was cast from a sol–gel of zinc acetate, and thermally converted to ZnO in air as described in literature [69]. A thin (10 nm) layer of PEIE has been shown in the past to improve contact by reducing the work function of a ZnO surface, and was prepared as reported [70]. The *J–V* characteristics of the films cast with no DIO in the standard and inverted device architecture are shown in Figure 6.

Devices cast from pure chlorobenzene achieved much higher efficiency in the inverted architecture than in the standard architecture (*J*_SC_ = 4.5 mA/cm^2^, *V*_OC _*=* 1.09 V, *FF* = 0.51, *PCE* = 2.5%). While the performance is still modest, there is a marked improvement in the shape of the *J–V* curve. The devices achieve high open circuit voltage with no sign of space charge build-up. All device parameters improve. While we cannot be completely sure the morphology of this film is identical as when it is in a standard architecture, this is a strong indication that the primary cause for the s-shape is indeed non-ideal vertical phase separation. Further SIMS analysis of the inverted cells and optimization of the film casting process in the inverted architecture may be the focus of future work.

Unfortunately, when the optimized 1.5% DIO condition was used to make inverted devices, the efficiency was lower than in a standard architecture (*J*_SC_ = 7.0 mA/cm^2^, *V*_OC _*=*0.73 V, *FF* = 0.47, *PCE* = 2.4%). These devices showed very high dark (leakage) current (Supporting Information File 1, Figure S10), which is likely a result of a change in morphology when casting atop ZnO instead of PEDOT; the films show significantly rougher surfaces (Supporting Information File 1, Figure S11). It is possible that through further optimization using the inverted architecture, we may be able to improve the top efficiency, however, that is beyond the scope of this work. We were satisfied to demonstrate that changing architectures does indeed eradicate the s-shape of the curve for devices cast from pure chlorobenzene, helping to further prove the hypothesis that the root of s-shaped *J–V* curves was indeed non-ideal vertical phase separation.

## Conclusion

A new molecular donor material, p-SIDT(FBTThCA8)_2_, was developed based on the inclusion of electron-withdrawing endcaps within a previously reported high-performance molecular framework. The structural modification had the desired effect of reducing the band gap for extended absorption in the visible spectrum while maintaining a low-lying HOMO level to achieve high *V*_OC_. The energy levels are nearly ideal match for incorporation into BHJ devices with the acceptor PC_71_BM, maximizing voltage and spectral coverage. Despite the structural similarity to previously reported materials, however, blends of p-SIDT(FBTThCA8)_2_ and PC_71_BM did not have device performance akin to its predecessors when processed in the same manner.

Specifically, when cast from chlorobenzene, the resulting *J–V* curve gives rise to a significant s-shape, resulting in extremely low *FF* and *PCE*. Through light intensity studies, the s-shape in the curve was attributed to the build-up of space charge. The use of DIO as a solvent additive helped to remove the s-shape character from the *J–V* curve and to improve the performance up to *PCE* = 3.2%. Analogous to what has been reported in literature, DIO helps to induce crystallinity of the p-SIDT(FBTThCA8)_2_ in the blend as evidenced by GIWAXS and a commensurate red shift in absorption. However, lack of crystallinity is not typically associated with the s-shape in the *J–V* curve seen when cast without additive.

Blends cast from chlorobenzene have reasonably high mobility, so a build-up of space charge simply due to an imbalance in carrier mobilities can likely be ruled out. Instead, the differences in curve shape are ascribed to changes in the vertical phase separation; when cast without additive there is a enrichment of PC_71_BM at the PEDOT:BHJ interface as evidenced by DSIMS. Subsequently, the low concentration of p-SIDT(FBTThCA8)_2_ at the anode likely leads to reduced surface recombination, a build-up of space charge and ultimately, and s-kink in the *J–V* curve. The inclusion of DIO helps to reduce the concentration of PC_71_BM at the anode improving surface recombination, and *J–V* characteristics. This is further evidenced by the elimination of the s-kink upon moving to an inverted structure. In fact, in the inverted structure the blend device gives a *V*_OC_ of 1.09 V, which is quite remarkable, considering the absorption profile extends out to 730 nm (1.69 eV). Such a small voltage loss between absorption onset and V_OC_ demonstrates the tremendous potential of this blend system. Although without further device engineering the performance of this materials system is not yet on par with the state of the art, the drastic change in curve shape is important in understanding the nature of solvent additives and their effects on solution processed BHJ devices.

## Supporting Information

File 1 Detailed experimental procedures with physical and chemical analysis of compounds and additional device characterization data.
