# Supplementary material for: Effects of solvent additive on “s-shaped” curves in solution-processed small molecule solar cells
Source: Beilstein J Org Chem. 2016 Nov 28;12:2543–55. doi: 10.3762/bjoc.12.249 (PMC5238578; doi:10.3762/bjoc.12.249)
Supplement: File 1 — Detailed experimental procedures with physical and chemical analysis of compounds and additional device characterization data. [file Beilstein_J_Org_Chem-12-2543-s001.pdf]

Supporting Information

for

Effects of solvent additive on "s-shaped" curves in solution-processed small molecule solar cells

John A. Love[1,2] *, Shu-Hua Chou [1,3], Ye Huang[1], Guillermo C. Bazan[1]* and Thuc-Quyen Nguyen[1]*

Address: [1]Center for Polymers and Organic Solids, University of California, Santa Barbara, California 93106, United States, [2]Institute of Physics and Astronomy, University of Potsdam, Potsdam-Golm 14476, Germany and [3]Department of Chemistry, National Taiwan University, Taipei, 10617, Taiwan

Email: John A. Love* - johnlove@uni-potsdam.de ; Guillermo C. Bazan* - bazan@chem.ucsb.edu; Thuc-Quyen Nguyen* - quyen@chem.ucsb.edu

*Corresponding author

Detailed experimental procedures with physical and chemical analysis of compounds and additional device characterization data

Table of Contents:

Materials and methods S3

Synthetic procedure and characterization S5

Molecular characterization........ S9
- Differential scanning calorimetry (DSC) S9
- Table of photophysical and thermal characteristics S9
- Cyclic voltammetry........ S10
- Table of electrochemical and theoretical energy levels S10

Device characterization S11

Light intensity dependent *J*–V curves S11
Grazing incidence wide angle X-ray scattering (GIWAXS) S11
Atomic force microscopy (AFM) of blend films in standard architecture S12
Hole mobility measurements S12
J–*V* characteristics of inverted solar cells processed with DIO S13
AFM of blend films in inverted architecture S14

References S14

Materials and methods

Materials. All chemicals and reagents were commercially available and used as received. Anhydrous tetrahydrofuran and toluene for chemical reactions were obtained by passing through solvent dispensing systems. 4,7-Dibromo-5-fluorobenzo[*c*][1,2,5]-thiadiazole was purchased from 1-Material Inc. and used without further purification as received. Benzo-[1,2-*b*:4,5-*b*]-bis-(2-bromo-4,4'-dihexyl-4*H*-silolo[3,2-*b*]-thiophene [1] and (*E*)-octyl-3-(5-bromothiophen-2-yl)-2-cyanoacrylate [2] were synthesized according to the literatures.

Nuclear magnetic resonance (NMR) spectroscopy. ^{1}H NMR were recorded on a Varian Unity Inova 500 MHz or 600 MHz spectrometer and ^{13}C NMR were performed on a Varian Unity Inova 500 MHz or Bruker Avance 800 MHz spectrometer at room temperature using deuterochloroform (or other given solvents) as internal reference. Chemical shifts are reported as δ values (ppm) relative to an internal tetramethylsilane (TMS) standard. 2D ^{1}H-^{1}H NOESY correlation experiments were acquired on a Varian Unity Inova 600MHz spectrometer at 25 ^{o}C for elucidating the fluorine regiochemistry of compound **2**.

Mass spectrometry (MS). Low-resolution field-desorption time-of-flight (FD-TOF) MS was performed on a Waters GCT Premier and high-resolution electrospray (ESI) MS was measured on a Waters Micromass QTOF2. All MS spectra were collected by the UCSB Department of Chemistry and Biochemistry Mass Spectrometry Facility.

Differential scanning calorimetry (DSC). The melting and crystallization temperatures were measured using a TA Instrument DSC (Model Q-2000) with a scan rate of 10 °C/min.

Cyclic voltammetry (CV). Solution electrochemical properties were performed using a CHI instrument model 730B in a three-electrode electrochemical cell with a glassy carbon working electrode, a Pt wire counter electrode, and a Ag wire pseudo reference electrode. CH_2Cl_2 was purified by distillation for cyclic voltammetry measurement. Redox potentials were measured in anhydrous CH_2Cl_2 with 0.1 M tetrabutylammonium hexafluorophosphate ($TBAPF_6$) as a supporting electrolyte, and product concentration was kept at 1 $mg \cdot mL^{-1}$. All potentials

reported here were calibrated with the ferrocene/ferrocenium redox couple (Fc/Fc^+) as internal standard. The corresponding HOMO and LUMO energy levels were acquired by correlating the onsets of oxidation and reduction to the Normal Hydrogen Electrode (NHE), assuming HOMO of Fc/Fc^+ to be 4.88 eV.

UV–visible absorption measurement. UV–vis spectra were recorded with a Beckman Coulter DU 800 series or a Perkin Elmer Lambda 750 spectrophotometer. All solution UV-Vis experiments were measured in $CHCl_3$. Neat films were prepared by spin-coating at a spin speed of 1500 rpm from 10 mg/mL $CHCl_3$ solutions atop pre-cleaned glass substrates. Films were annealed on a hot plate for 2 minutes at given temperatures and cooled to room temperature for absorption measurements.

Solubility measurements. Prepared the calibration curve by measuring the absorbance of different solutions in $CHCl_3$ with a known concentration, and plotting λ_{max} vs. concentration, a linear relationship was observed. An over-saturated $CHCl_3$ solution was stirred vigorously for 48 hours at room temperature. The slurry was then filtered through a 0.45 μm PTFE filter. The filtrate was assumed to be a saturated solution. A 100 μL aliquot was then diluted to 50 mL with $CHCl_3$. The UV–vis absorption spectrum was obtained and the concentration was determined using its standard calibration curve.

DFT theoretical calculations. The molecular geometry and photophysical properties of p-SIDT(FBTThCA8)$_2$ at the molecular level were performed by Gaussian 09 program, in which the conformation of ground state and HOMO/LUMO orbitals were optimized by the density functional theory (DFT) using Beck's three-parameterized Lee-Yang-Parr exchange functional (B3LYP) with 6-311G(d,p) basis sets.

GIWAXS measurement. Grazing Incidence Wide Angle X-ray Scattering (GIWAXS) patterns were collected at the Stanford Synchrotron Radiation Lightsource (SSRL) beamline 11-3 with an X-ray wavelength of 0.9752 Å, at a 40 cm sample to detector distance at an incident angle of 0.12^o. Samples were probed under a helium environment to minimize beam damage and reduce diffuse scattering. The measurements were calibrated using a LaB6

standard. Thin films were prepared by spin-coating from chloroform solution (10 mg/mL) atop silicon substrates at 1000 rpm.

Synthetic procedure and characterization

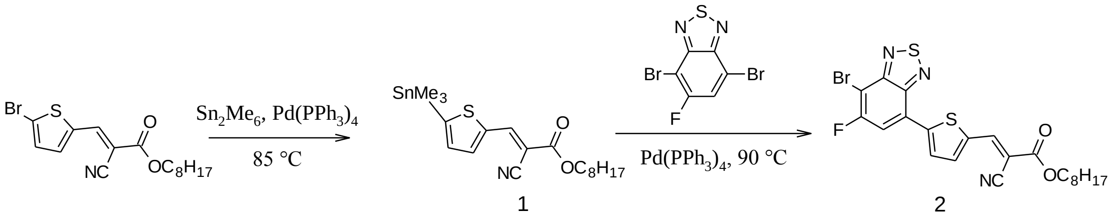

Synthesis of (E)-octyl 3-(5-(7-bromo-6-fluorobenzo[c][1,2,5]thiadiazol-4-yl)thiophen-2-yl)-2-cyanoacrylate (**2**). In a N_2 filled glove box, hexamethylditin (982.8 mg, 3.0 mmol), (*E*)-octyl 3-(5-bromothiophen-2-yl)-2-cyanoacrylate (740.6 mg, 2.0 mmol), $Pd(PPh_3)_4$ (115.5 mg, 0.1 mmol), and dry toluene (20 mL) were combined in a 50 mL round bottle and then sealed. The reaction mixture was stirred and heated at 85 °C for 18 hours. After cooling to room temperature, the solvent and excess amounts of hexamethylditin were removed by rotary evaporation. The crude material (*E*)-octyl 2-cyano-3-(5-(trimethyl-stannyl)thiophen-2-yl)acrylate (**1**) was used for the following procedure without further purification. A 50 mL round bottle was charged with crude **1**, 4,7-dibromo-5-fluoro-benzo[*c*][1,2,5]-thiadiazole (592.7 mg, 1.9 mmol), $Pd(PPh_3)_4$ (87.8 mg, 0.076 mmol) and dry toluene (30 mL) in a N_2 filled glove box. The reaction mixture was then sealed and stirred at 90 °C for 3 days. Upon cooling, the solvent was removed in vacuo. The crude product was purified by column chromatography on silica gel with gradient eluent from CH_2Cl_2/hexane (v/v, 1:3) to CH_2Cl_2/hexane (v/v, 1:1). The product was washed with MeOH and then dried under vacuum overnight. The desired product **2** was obtained as yellow solid (425 mg, 42.8%). ^{1}H NMR (600 MHz, $CDCl_3$, δ): 8.34 (s, 1H), 8.22 (d, J = 6 Hz, 1H), 7.89 (d, J = 6 Hz, 1H), 7.86 (d, J = 12 Hz, 1H), 4.32 (t, J = 6 Hz, 2H), 1.77 (q, J = 7.5 Hz, 2H), 1.45-1.41 (m, 2H), 1.34-1.28 (m, 8H), 0.89 (t, J = 6 Hz, 3H). ^{13}C NMR (125 MHz, $CDCl_3$, δ): 162.7, 161.6, 159.5, 154.4, 154.3, 148.7, 145.9, 138.0, 137.8, 129.7, 125.8, 118.0, 117.7, 115.8, 100.5, 99.3, 99.1, 67.1, 31.9, 29.4, 29.3, 28.7, 26.0, 22.8, 14.3. HRMS (ESI-TOF) *m/z*, calcd for $C_{22}H_{21}BrFN_3NaO_2S_2$ $[M+Na]^+$: 544.0140; found: 544.0118.

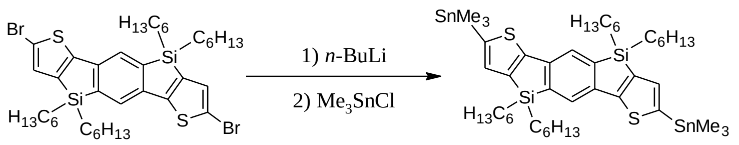

3

Synthesis of benzo[1,2-b:4,5-b]bis(2-trimethylstannyl-4,4'-dihexyl-4H-silolo[3,2-b]-thiophene) **(3)**. *n*-BuLi (1.6 M in hexane, 1.4 mL, 2.3 mmol) was injected dropwise via a syringe into a solution of benzo[1,2-*b*:4,5-*b*]-bis-(2-bromo-4,4'-dihexyl-4*H*-silolo[3,2-*b*]-thiophene (793 mg, 1.0 mmol) in anhydrous THF (100 mL) at −78 °C under N_2. The mixture was stirred at –78 °C for 10 min and then a solution of trimethyltin chloride (498.2 mg in 3.5 mL dry THF, 2.5 mmol) was added quickly in one portion at −78 °C. The reaction was stirred at room temperature for another 4 hours. Water was added to the reaction mixture and extracted with hexanes. The combined organic layer was dried over anhydrous $MgSO_4$ and the solvent was removed in vacuo. The solid was precipitated out in iced MeOH solution and filtered. Compound **3** was collected as pale yellow solid (950 mg, 98.9%). ^{1}H NMR (600 MHz, $CDCl_3$, δ): 7.57 (s, 1H), 7.16 (s, 1H), 1.44-1.39 (m, 4H), 1.32-1.21 (m, 12H), 0.98-0.89 (m, 4H), 0.85-0.83 (m, 6H), 0.4 (s, 9H). ^{13}C NMR (125 MHz, $CDCl_3$, δ): 163.3, 142.4, 142.2, 140.7, 138.8, 138.0, 125.7, 33.2, 31.6, 24.3, 22.8, 14.3, 12.4, -7.9. LRMS (FD-TOF) *m/z,* calcd for $C_{44}H_{74}S_2Si_2Sn_2$ $[M]^+$: 962.28; found: 962.28.

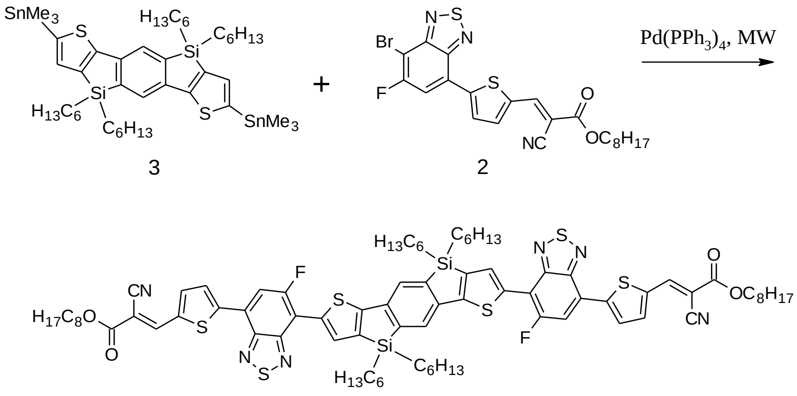

p-SIDT(FBTThCA8)$_2$

Synthesis of **p-SIDT(FBTThCA8)$_2$**. To a 20 mL microwave vial was added compound **2** (493 mg, 0.94 mmol), compound **3** (432 mg, 0.45 mmol), $Pd(PPh_3)_4$ (26 mg, 0.023 mmol), and dry toluene (13 mL) in a N_2 filled glove box. The reaction vial was sealed and loaded in a

Biotage microwave reactor with conditions as follows: 100 °C for 5 minutes, 120 °C for 5 minutes, 140 °C for 10 minutes and 170 °C for 50 minutes. Upon cooling, the volatiles were removed in vacuo. The crude product was purified through silica gel column with gradient eluent from $CHCl_3$/hexane (v/v, 1:1) to pure $CHCl_3$. Purification by column chromatography was carried out several times to ensure purity for device fabrication. The solid was reprecipitated and filtered with DCM/MeOH, DCM/pentane and DCM/ethylacetate sequentially. **p-SIDT(FBTThCA8)$_2$** (550 mg, 80.6%) was then obtained as bronze-colored solid after dried under vacuum for 24 hours. ^{1}H NMR (500 MHz, $CDCl_3$, δ): 8.41 (s, 2H), 8.33 (s, 2H), 8.24 (d, *J* = 5 Hz, 2H), 7.95 (d, *J* = 15 Hz, 2H), 7.88 (d, *J* = 5 Hz, 2H), 7.77 (s, 2H), 4.31 (t, *J* = 5 Hz, 4H), 1.80-1.74 (m, 4H), 1.53-1.46 (m, 8H), 1.45-1.41 (m, 4H), 1.39-1.25 (m, 40H), 1.12-1.01 (m, 8H), 0.91-0.84 (m, 18H). ^{13}C NMR (200 MHz, $CDCl_3$, δ): 163.0, 160.9, 160.8, 159.0, 157.7, 153.4, 153.3, 149.8, 147.4, 147.3, 146.1, 143.2, 142.9, 140.7, 138.2, 137.1, 134.7, 134.6, 134.1, 134.0, 129.0, 126.0, 123.0, 122.9, 118.9, 118.8, 116.1, 114.4, 114.3, 99.5, 67.0, 33.2, 32.0, 31.7, 29.4, 29.3, 28.8, 26.0, 24.3, 22.9, 22.8, 14.3, 14.2, 12.4. LRMS (FD-TOF) *m/z*, calcd for $C_{82}H_{98}F_2N_6O_4S_6Si_2$ $[M]^+$: 1516.55; found: 1516.46.

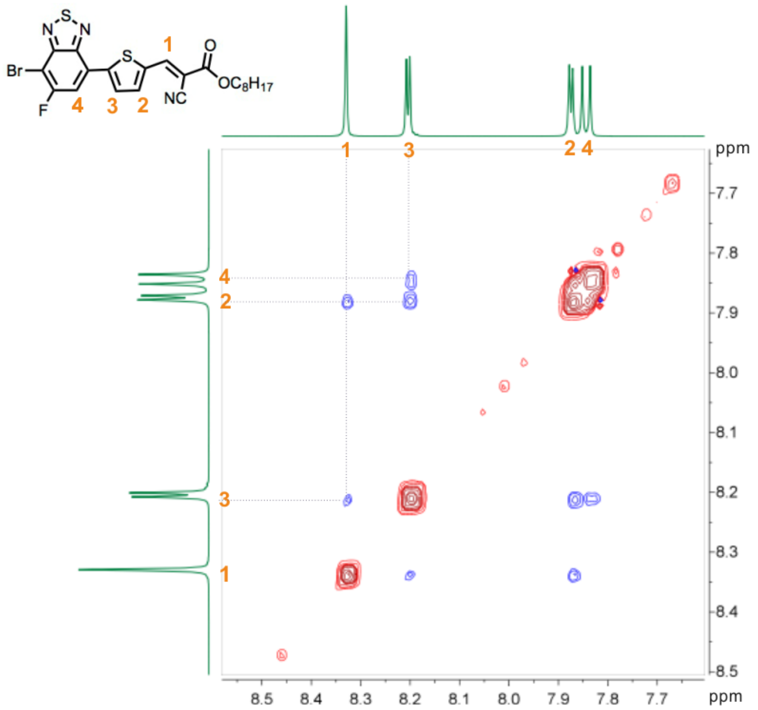

Figure S1: **2D ^{1}H-^{1}H NOESY spectrum** of compound **2** in $CDCl_3$ expands to the selected aromatic region and the assignment of its regiochemistry.

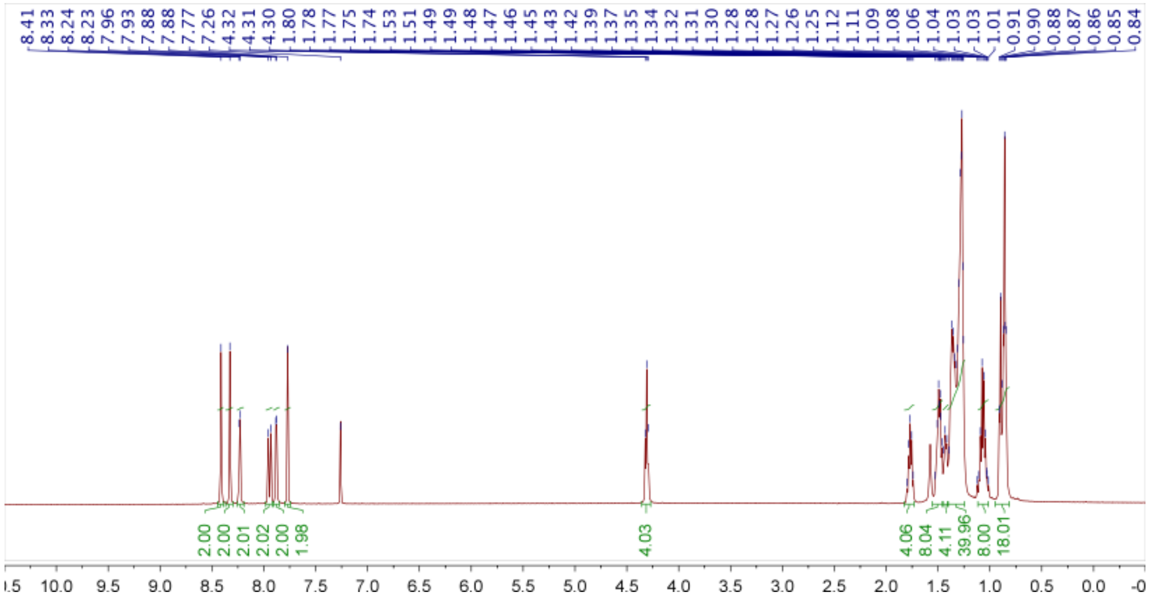

Figure S2. **^{1}H NMR spectrum** of p-SIDT(FBTThCA8)$_2$ in $CDCl_3$.

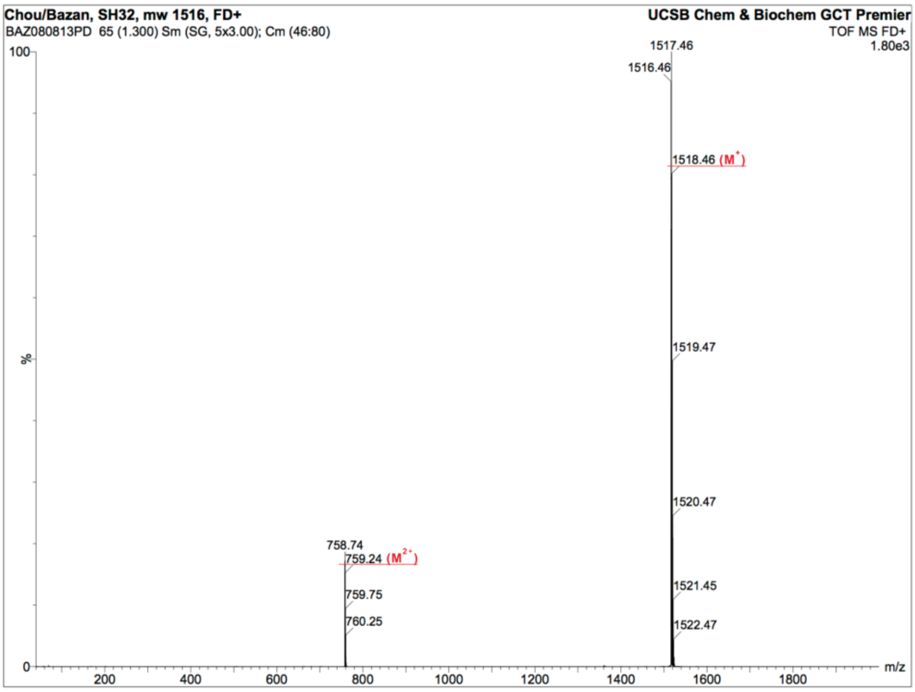

Figure S3: FD-TOF MS of p-SIDT(FBTThCA8)$_2$, showing double-charged species (M^{2+}).

Molecular characterization

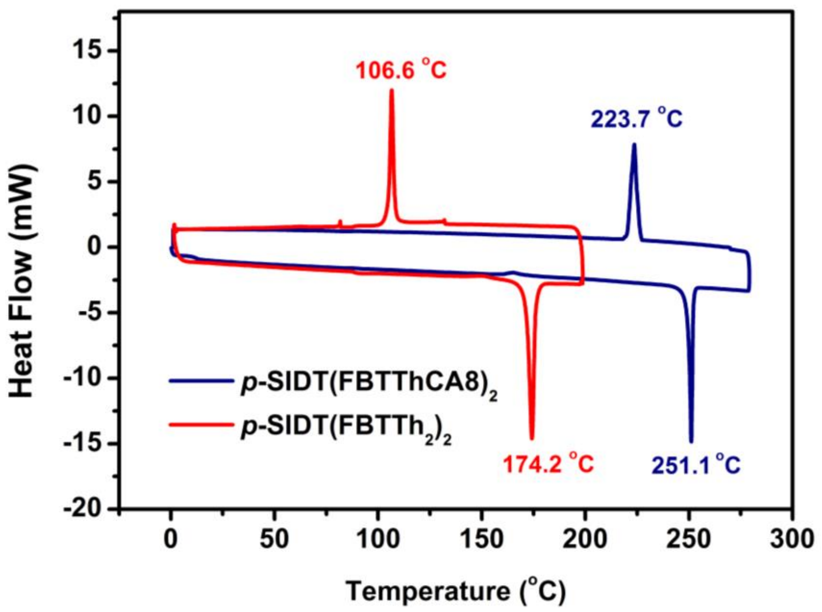

Figure S4: DSC thermograms showing melting (T_m) and crystallization (T_c) temperatures of p-SIDT(FBTThCA8)$_2$ and p-SIDT(FBTTh$_2$)$_2$.

Table S1: Photophysical and thermal characteristics of p-SIDT(FBTThCA8)$_2$ and p-SIDT(FBTTh$_2$)$_2$.

Molecule	solution absorption[a]		film absorption		thermal transitions
	λ_{max} (nm)	ε (λ_{max}) ($M^{-1}cm^{-1}$)	λ_{max} (nm)	ΔE^{opt} (eV)[b]	T_m/T_c (°C)
p-SIDT(FBTThCA8)$_2$	603	96630	650	1.65	223.7/ 251.1
p-SIDT(FBTTh$_2$)$_2$	571	65000	610	1.93	106.6/ 174.2

[a] Measured in $CHCl_3$ at room temperature; [b] obtained from the absorption onsets in the films.

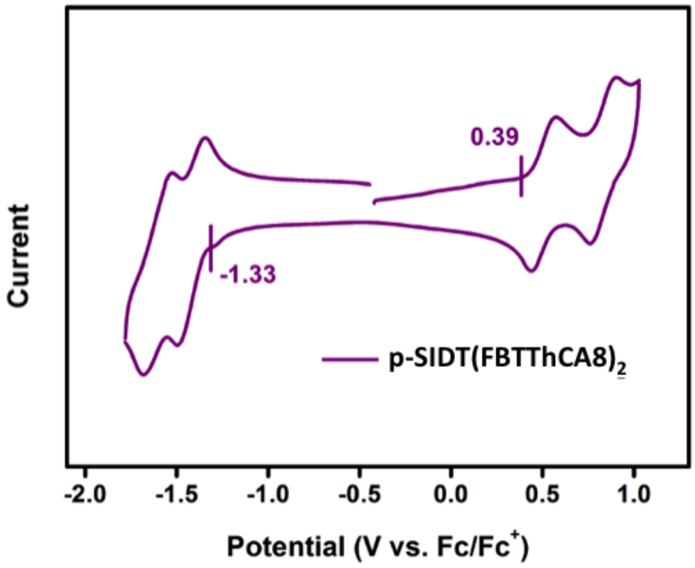

Figure S5: Cyclic voltammetry of p-SIDT(FBTThCA8)$_2$.

Table S2: Electrochemical and theoretical energy levels of p-SIDT(FBTThCA8)$_2$ and p-SIDT(FBTTh$_2$)$_2$.

Molecule	Energy levels	
	$E_{HOMO}/E_{LUMO}/\Delta E^{CV}$ (eV)[a]	$E_{HOMO}/E_{LUMO}/\Delta E^{DFT}$ (eV)[b]
p-SIDT(FBTThCA8)$_2$	−5.27/−3.55/1.72	−5.43/−3.53/1.90
p-SIDT(FBTTh$_2$)$_2$	−5.21/−3.36/1.85	−4.97/−2.96/2.01

[a]Determined by CV experiment; [b]calculated by the DFT theoretical simulation of optimized geometry.

Device characterization

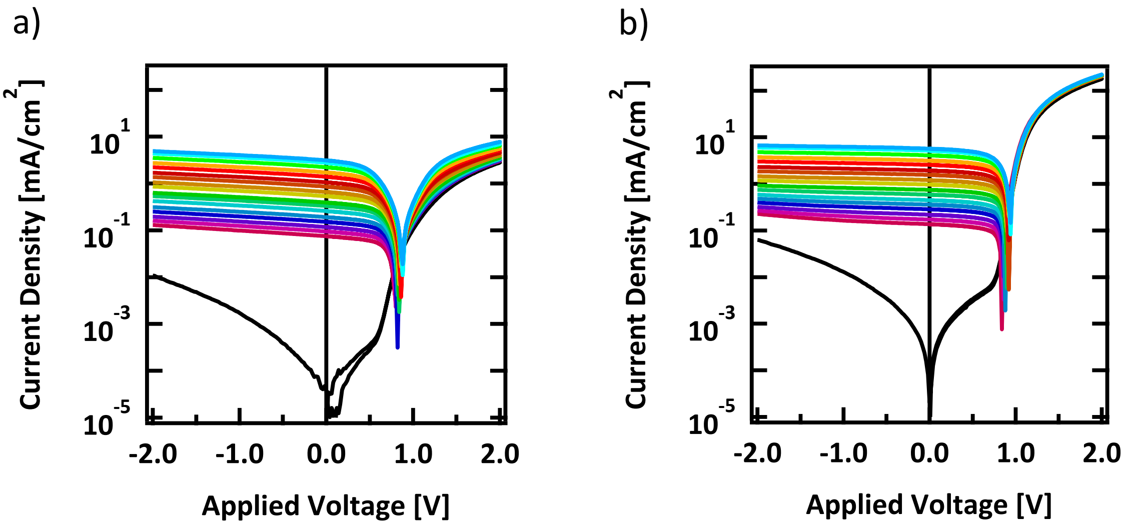

Figure S6: Light intensity dependence of p-SIDT(FBTThCA8)$_2$:PC$_{71}$BM devices cast a) from pure chlorobenzene and b) with 1.5% DIO ranging from 100 mw/cm^2 (blue) down to 2 mW/cm^2 (pink) showing extremely low dark current (black).

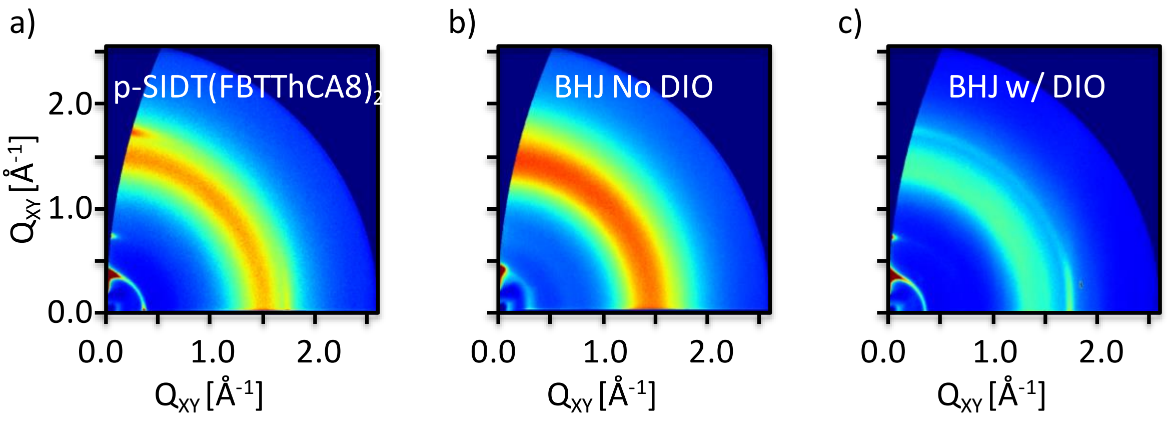

Figure S7: Grazing incidence wide angle x-ray scattering profiles of BHJ films cast with and b) with DIO showing intensity as a function of both reciprocal distance, Q and angle, χ.

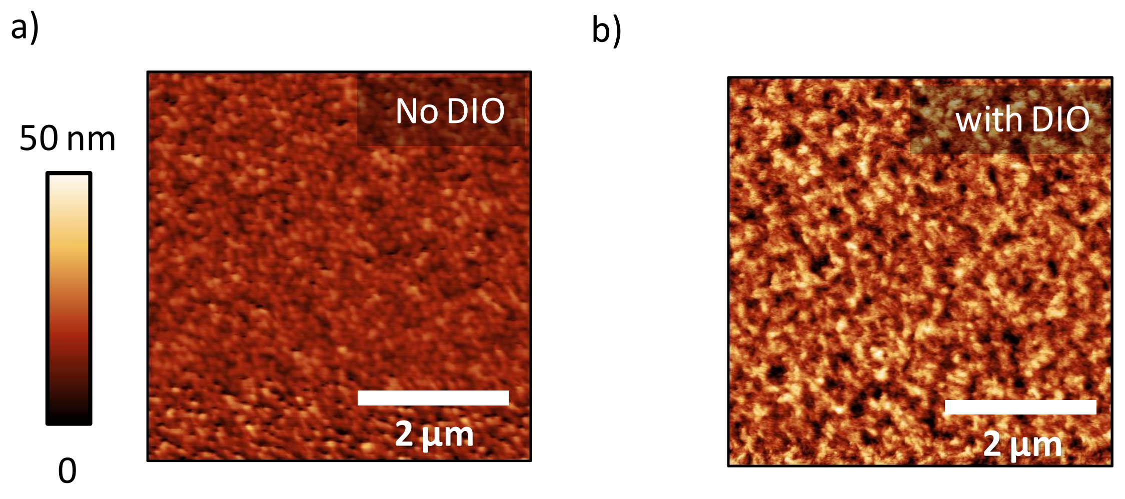

Figure S8: AFM topography images of p-SIDT(FBTThCA8)$_2$:PC$_{71}$BM devices cast (a) without and (b) with 1.5% DIO atop PEDOT.

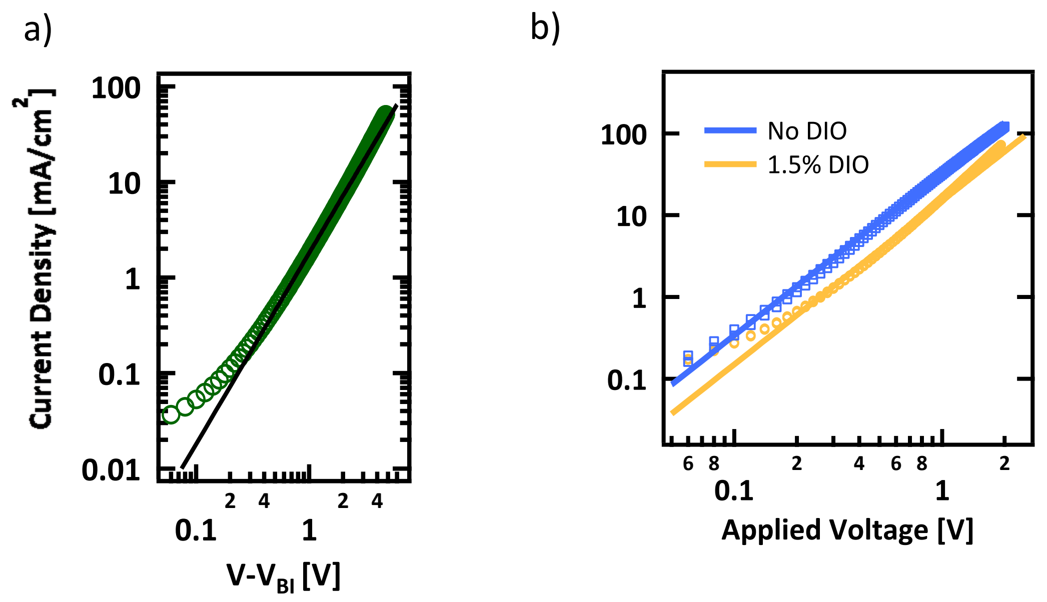

Figure S9: Hole mobility fits to single carrier devices of a) neat p-SIDT(FBTThCA8)$_2$ and b) p-SIDT(FBTThCA8)$_2$:PC$_{71}$BM blends cast from pure chlorobenzene (yellow) and 1.5 % DIO (blue). Mobilities for neat, as-cast, and 1.5% DIO layers were found to be 2.0×10^{-4}, 4.5×10^{-5}, and 9.2×10^{-5} cm^2/Vs respectively.

Hole only devices were fabricated for pristine p-SIDT(FBTThCA8)$_2$ and BHJ blends according to the structure ITO/PEDOT/active layer/MoO_3/Ag. The *J*–*V* characteristics of the

devices were measured in the dark. Injection current from the MoO_3 top contact was fit according to the Mott–Gurney Law for space charge limited current:

$$J = \frac{9}{8}\varepsilon_0\varepsilon_r\mu_p\frac{V^2}{L^3}$$

where ε_0 is the vacuum permittivity, ε_r is the relative dielectric of the active layer (assumed to be 3 in all cases), μ_p is the hole mobility V is the applied voltage minus a small built in voltage, and L is the active layer thickness, which for neat p-SIDT(FBTThCA8)$_2$ was varied between 250 and 350 nm and for BHJ blends were kept at 120 nm as in solar cells.

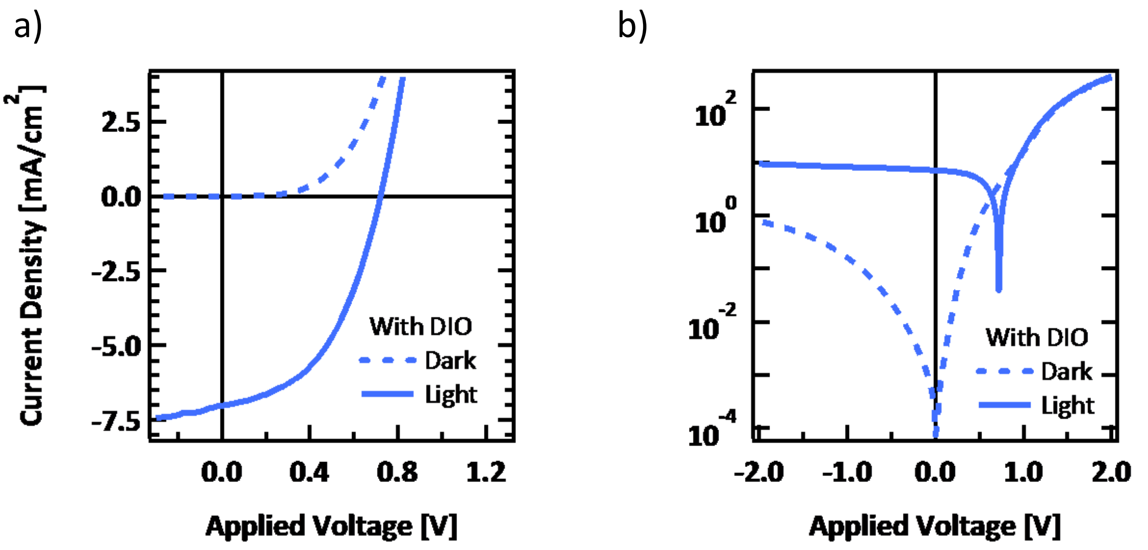

Figure S10: *J–V* characteristics of inverted solar cells cast with 1.5% DIO.

When the p-SIDT(FBTThCA8)$_2$:$PC_{71}BM$ layer was cast with 1.5% DIO in the inverted architecture of ITO/ZnO/PEIE/BHJ/MoO_3/Al devices showed large dark current as shown above in Figure S10. Unfortunately this leads to extremely low V_{OC} and *FF* leading to low performance despite improved photocurrent compared to the standard architecture.

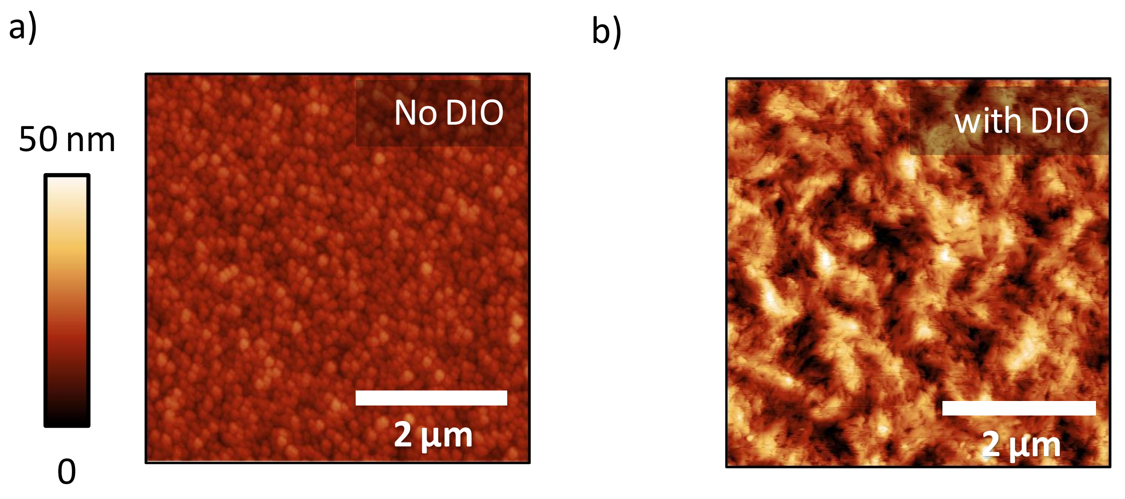

Figure S11: AFM topography images of p-SIDT(FBTThCA8)$_2$:PC$_{71}$BM devices cast (a) without and (b) with 1.5% DIO atop ZnO/PEIE (inverted architecture).

The high dark current observed for 1.5% DIO inverted devices likely is due to a change in morphology caused by casting atop the different substrates.. When the p-SIDT(FBTThCA8)$_2$:PC$_{71}$BM is cast with 1.5% DIO on ZnO/PEIE substrates (Figure S11 b), the roughness and size scale of the features increases dramatically. Films cast without additive, from pure chlorobenzene, show smoother, surfaces (RMS roughness $<$ 3 nm) regardless of underlying substrate.

References

[1] J. A. Love, I. Nagao, Y. Huang, M. Kuik, V. Gupta, C. J. Takacs, J. E. Coughlin, L. Qi, T. S. van der Poll, E. J. Kramer, A. J. Heeger, T.-Q. Nguyen, G. C. Bazan, *J. Am. Chem. Soc.* **2014**, DOI 10.1021/ja412473p

[2] Y. Lin, Z.-G. Zhang, H. Bai, Y. Li, X. Zhan, *Chem. Commun.* **2012**, *48*, 9655.
